# National Thalassemia Prevention Campaign: The Talotır Project

**DOI:** 10.4274/tjh.2012.0121

**Published:** 2013-03-05

**Authors:** Duran Canatan, Yeşim Aydınok, Yurdanur Kılınç, Zeynep Karakaş, İlgen Şaşmaz, Hilmi Apak, Nazan Sarper

**Affiliations:** 1 Mediterranean Blood Diseases Foundation, Hemoglobinopathy Diagnosis Center, Antalya, Turkey; 2 Ege University, Department of Pediatric Hematology, İzmir, Turkey; 3 Çukurova University, Department of Pediatric Hematology, Adana, Turkey; 4 İstanbul University, Çapa Medical Faculty, Department of Pediatric Hematology, İstanbul, Turkey; 5 İstanbul University, Cerrahpaşa Medical Faculty, Department of Pediatric Hematology, İstanbul, Turkey; 6 Kocaeli University, Department of Pediatric Hematology, Kocaeli, Turkey

**Keywords:** Thalassemia, prevention, Campaign

**To the Editor**

Thalassemia and abnormal hemoglobins are a serious health problem throughout the world. The first preventive and educational studies were begun in Italy in the 1970s by the World Health Organization [1]. In Turkey, very important steps for treating thalassemia have been taken since 2000. The Turkish National Hemoglobinopathy Council (TNHC) was created to combine all relevant centers, foundations, and associations into one organization together with the Ministry of Health (MOH) in 2000. New regulations were issued by the MOH and TNHC in 2002. Data on patients with thalassemia and hemoglobinopathies were collected from all 81 provinces in Turkey and revealed a map of hemoglobinopathy in Turkey in 2002. A hemoglobinopathy control program (HCP) was begun in 33 provinces in 2003 [2]. The Thalassemia Federation was established as a civil society organization in 2005. The National Thalassemia Prevention Campaign (NTPC) was organized for public education and support of the HCP by the Thalassemia Federation. The aim of this campaign was to educate different parts of the population and raise awareness about thalassemia and hemoglobinopathies in Turkey. 

The Thalassemia Federation arranged an assembly to plan and design this project with representatives from the MOH, Ministry of the Interior, Ministry of Education, and Ministry of Religious Affairs before the NTPC. Master slides including both public and health workers’ education programs were prepared by a scientific committee. A long-term project called Talotır was specially designed for education and training. 

A total of 23 provinces in Thrace and the western and southern Anatolia regions, including a population of about 50 million with high incidences of thalassemia, were chosen for Talotır. The target population for education primarily comprised doctors, other healthcare staff, teachers, students, officials, religious leaders, and patients and their families, in addition to the public.

The Talotır educational tour was launched at Taksim Square in İstanbul on 8 May 2007, World Thalassemia Day, and concluded in Antalya on 23 December of that year. 

As a result, a total of 62,682 people were educated in those 8 months in 23 provinces. This represents 0.12% of the total population living in those 23 provinces being educated about thalassemia, with raised awareness in the broader population by the press and media in Turkey.

According to reports by the MOH, while premarital screening tests were done in 30% of all marriages in 2003, this rate reached 81% in 2008. As the result of these screenings, the number of infants born with thalassemia was decreased by 87% in 2008. The objectives of the HCP were to control and ultimately to eradicate thalassemia and hemoglobinopathies in Turkey. The success of preventive programs depends on educational programs and campaigns [3]. This campaign provided very important support to the HCP in Turkey, because a total of 62,682 people (0.12% of the total population), including healthcare workers, students, teachers, officials, religious leaders, and others, were educated about thalassemia and hemoglobinopathies.

In conclusion, educational programs are very important for prevention thalassemia and hemoglobinopathy. Campaigns and educational programs will continue for a Turkey without thalassemia in next years.

**Acknowledgments**: We would like to thank all trainers of the educational program, the representatives of the Ministry for planning, and Novartis Oncology for financial support.

**Conflict of Interest Statement**

The authors of this paper have no conflicts of interest, including specific financial interest, relationship, and/or affiliations relevant to the subject matter or materials included.
